# NETTAB 2012 on "Integrated Bio-Search"

**DOI:** 10.1186/1471-2105-15-S1-S1

**Published:** 2014-01-10

**Authors:** Paolo Romano, Frédérique Lisacek, Marco Masseroli

**Affiliations:** 1 Bioinformatics, IRCCS AOU San Martino - IST National Cancer Research Institute, Genoa, I-16132, Italy; 2 Proteome Informatics Group, SIB Swiss Institute of Bioinformatics, 1211 Geneva 4, Switzerland; 3 Section of Biology, University of Geneva, 1211 Geneva 4, Switzerland; 4 Dipartimento di Elettronica, Informazione e Bioingegneria, Politecnico di Milano, Milano, I-20133, Italy

## Abstract

The NETTAB 2012 workshop, held in Como on November 14-16, 2012, was devoted to "Integrated Bio-Search", that is to technologies, methods, architectures, systems and applications for searching, retrieving, integrating and analyzing data, information, and knowledge with the aim of answering complex bio-medical-molecular questions, i.e. some of the most challenging issues in bioinformatics today. It brought together about 80 researchers working in the field of Bioinformatics, Computational Biology, Biology, Computer Science and Engineering. More than 50 scientific contributions, including keynote and tutorial talks, oral communications, posters and software demonstrations, were presented at the workshop. This preface provides a brief overview of the workshop and shortly introduces the peer-reviewed manuscripts that were accepted for publication in this Supplement.

## NETTAB Workshops

NETTAB Workshops are a series of International meetings on "Network Tools and Applications in Biology" held annually in Italy [1]. They are aimed at introducing participants to the most promising among those innovative Information and Communication Technologies (ICTs) that are being applied to the biomedical application domain. Workshops include many focused sessions which are devoted to tools, systems, applications and perspectives. Keynote lectures introduce the sessions' topics and are followed by presentations selected from among the submitted contributions after peer review by members of the Scientific Committee. Discussion is a key factor, both within sessions and in a special Panel Discussion. Tutorials and poster sessions usually complete the agenda of the NETTAB workshops.

Each year, the workshop is focused on a different technology or domain. Since 2001, many different topics, often related to data integration issues, were discussed, thus reflecting the actual evolution of ICT tools and platforms in the last decade [2]. These included, e.g., Standardization for data integration (Genoa, 2001), Multi-agent systems (Bologna, 2002), Scientific workflows (Naples, 2005), Grid and Web Services (Santa Margherita di Pula, 2006), Semantic Web (Pisa, 2007), Collaborative research and development (Catania, 2009), Biological wikis (Naples, 2010) and Clinical Bioinformatics (Pavia, 2010).

## The twelfth NETTAB Workshop: NETTAB 2012

The NETTAB 2012 workshop, the twelfth in the series, was held in Como, Italy, on November 14-16, 2012. It was organized by Marco Masseroli, Politecnico di Milano, Milano, Paolo Romano, Cancer Comprehensive Center and University Hospital San Martino IST, Genova, and Frédérique Lisacek, Swiss Institute of Bioinformatics, Geneva. Its rationale is based on the consideration that the data deluge of the current post-genomic era is providing scientists with potentially very valuable but often inaccessible information. It is indeed difficult to find and extract from the high-throughput omics data those information that are most reliable, specific and most related to the biological or biomedical questions to be answered. Such questions are increasingly complex and they often simultaneously regard many heterogeneous aspects of an organism, tissue, or cell, and the role of their biomolecular entities. Several of these questions can be addressed only by comprehensively searching different types of data, which generally are distributed in many heterogeneous sources. Usually, scientists explore these data by using the individual search services and tools available on the Internet and they then struggle to combine the essential information in order to answer their global questions. In this context, moreover, quality and consistency checking is a central issue that should be addressed.

Searching and combining numerous open and linked data and algorithmic sources has the potential of reshaping the scenario of current bioinformatics applications, going beyond the capabilities of conventional tools, Web services and existing search engines. Yet, it also presents new technological challenges. Solving data integration and automatic extraction problems requires new solutions, including the use of universal Uniform Resource Identifiers (URIs), efficient indexing, partial or approximate value matching, rank aggregation, continuous or push-based search, exploratory methods and context-aware paradigms, collaborative and social search; it also needs building new efficient information retrieval approaches, based on automation of workflows, that may contribute to new "good practices" in data searching, retrieval and integration, with the specific goal of ensuring quality of procedures, as well as their reproducibility coupled with efficiency and efficacy.

On these premises, then, the NETTAB 2012 workshop has been focused on "Integrated Bio-Search", which includes all aspects that relate to technologies, methods, architectures, systems and applications for searching, retrieving, integrating and analyzing data, information, knowledge, infrastructures, services and tools that are required to answer complex bio-medical-molecular questions.

The Call for abstracts attracted 34 submissions for oral communications. All submissions underwent peer review by members of the Scientific Committee that selected 12 oral communications, seven short oral communications, and three technological communications from industry; 29 posters were also presented at the workshop. The Proceedings were published by the EMBnet.journal [3].

Three keynote talks were given. Erik Bongcam-Rudloff, from the Swedish University of Agricultural Sciences and the Uppsala University, gave a talk on "Integration and analysis of multi-type high-throughput data for biomolecular knowledge discovery". "Semantics based biomedical knowledge search, integration and discovery" was the title of the lecture given by Barend Mons, Leiden University Medical Center and Netherlands Bioinformatics Center. Finally, Eric Neumann, PanGenX and Clinical Semantics Technologies, gave a talk on "Clinical and genomic data integration in support of biomedical research and clinical practice".

Two appreciated tutorials were also given by Alexander Kel, GeneXplain GmbH and Institute of Chemical Biology and Fundamental Medicine SBRAS, on "Multi-scale data integration and virtual exploration from promoters, through networks to drug targets", and by Katy Wolstencroft, University of Manchester, who spoke about "The Taverna Workbench: Integrating and analysing biological and clinical data with computerised workflows". It is noteworthy that the Web site of the workshop includes the video recording of almost all of oral presentations [4].

## Selection of best papers

Twenty nine (29) papers were submitted for publication in this BMC Bioinformatics Supplement after the conference. An Editorial Board was formed, including all members of the NETTAB 2012 Scientific Committee. Associated Editors were:

• Francisco Azuaje, Centre de Recherche Public de la Santé, Luxembourg

• Olivier Bodenreider, US National Library of Medicine, USA

• Mario Cannataro, University of Catanzaro "Magna Graecia", Italy

• Marie-Dominique Devignes, CNRS, University of Lorraine, Nancy, France

• Christine Froidevaux, Université Paris-Sud, France

• Carole Goble, University of Manchester, United Kingdom

• Nicolas Le Novère, European Bioinformatics Institute, United Kingdom

• Ulf Leser, Humboldt University, Germany

• Frédérique Lisacek, Swiss Institute of Bioinformatics, Switzerland

• Paolo Magni, University of Pavia, Italy

• Roberto Marangoni, University of Pisa, Italy

• Marco Masseroli, Politecnico di Milano, Italy

• Paolo Missier, Newcastle University, United Kingdom

• Heiko Muller, Italian Institute of Technology, Italy

• Horacio Pérez-Sánchez, University of Murcia, Spain

• Paolo Romano IRCCS AOU San Martino IST, Italy

• Patrick Ruch, University of Applied Sciences, Switzerland

• Neil Sarkar, University of Vermont, USA

Each Associate Editor managed the reviewing process for one or two papers, according to his/her expertise in workshop topics. Three international level referees were selected for each submission. Overall, 54 referees from 11 different countries were involved in the selection of papers. A two step peer review procedure was adopted: some of the authors were invited to submit a revised version of their paper, according to the referees' comments, when it wasn't neither accepted nor rejected at the first step. The Associated Editors made a global assessment for papers assigned to each of them and provided the final recommendation for each paper. At the end of this process, 14 papers were proposed and are now included in this Supplement, and one more paper was proposed for publication in another journal.

## A short presentation of selected papers

Workshop topics included four main areas. The first area relates to data integration. It includes syntactic and semantic methods and algorithms for biological and clinical data and knowledge integration, information and knowledge retrieval, data and knowledge query, data, information and knowledge extraction, and data and knowledge mining. The second area refers to new and optimized technologies for data management. It includes federated databases, data warehouses, and triple stores. It also includes topics as biomedical terminologies and ontologies, systems' interoperability, natural language processing, and scientific workflow processing. Tools and platforms for molecular data management and storage, deep sequencing analysis, omics data computing, search computing, decision support, and clinical bioinformatics characterize the third topic area. The fourth area includes examples of applications of these methods, technologies and tools in different biomedical domains, such as biomedical knowledge assessment, integration, discovery and validation, drug design, diagnosis and prognosis support, and personalized medicine.

Masseroli, Mons et al. present some of the challenges and trends for the integration, search and processing of biological information [5]. Starting from the need for adopting common data models and for community driven, re-usable efforts, the role of large scale international research infrastructures and of public-private partnerships targeted to addressing the complex challenges of data intensive science is stressed. Some crucial social aspects are also discussed, as well as an open business model for bioinformatics which could be able to reduce duplication of efforts.

The paper by Masseroli, Picozzi et al. "Explorative search of distributed bio-data to answer complex biomedical questions" [6] presents the Bio-SeCo system, a platform dedicated to answer complex biomedical questions by combining different heterogeneous services and providing global, homogeneous results, thus facilitating navigation among distributed biomedical data and answering queries involving several kinds of data.

The paper by Pio, Malerba et al. "Integrating microRNA target predictions for the discovery of gene regulatory networks: a semi-supervised ensemble learning approach" [7] presents a machine learning based approach for the combination of different algorithms for the prediction of relationships between mRNA and miRNA, which is able to optimize the discovery of miRNA-mRNA regulatory networks.

In the paper "ProphNet: A generic prioritization method through propagation of information" [8], Martínez, Cano et al. propose a novel network-based method for the prioritization of a set of entities that is able to integrate an arbitrary number of interrelated biological entities, thus overcoming current limitations of prioritization tools.

Cremaschi, Rovida et al. are the authors of "CorrelaGenes: A new tool for the interpretation of the human transcriptomes" [9]. This paper presents a new approach and tool for mining public gene expression profiles from the Gene Expression Omnibus (GEO) system that couples association rules and χ^2 ^test. This tool is also able to make a great number of GEO expression data sets searchable.

In their paper "Reducing bias in RNA sequencing data: a novel approach to compute counts" [10], Finotello, Lavezzo et al. describe maxcounts, a novel approach for measuring exon expression levels from RNA-Seq data, defined as the maximum number of counts among the positions of an exon, that aims at a more accurate estimation of expression levels from RNA-Seq data. A comparison with a standard approach, using three different data sets and considering several criteria, is also presented.

The paper "AnnotateGenomicRegions: A Web application" [11] by Zammataro, De Molfetta et al. describes a simple, but fast and effective, Web application that accepts genomic regions as input, downloads genome annotations, both overlapping and neighbouring, from the Genome Browser, including RefSeq transcripts, EnsEMBL transcripts, all_mrna transcripts, CpG islands and promoter regions of transcripts, and makes them available through both a Web site and a Web API. Being available as a Web interface, AnnotateGenomicRegion is user-friendly and scales well with respect to the load.

Campbell, Ranzinger et al. diagnose the causes of the slow development of glycobioinformatics and the difficulties encountered in defining adequate formats for representing complex carbohydrates in their paper "Toolboxes for a standardised and systematic study of glycans" [12]. The paper strongly suggests the integration of glycomics in the -omics landscape to better understand biological processes and it highlights the necessary steps to achieve this goal.

In the paper "A tool for mapping Single Nucleotide Polymorphisms using Graphics Processing Units" [13], Manconi, Orro et al. present a tool which maps a short sequence of SNP against a DNA sequence to find its physical position in that sequence. The tool does not provide an original algorithm, but it leverages on three existing software applications. The integration of existing software to solve a concrete problem, however, is a valuable solution for many biological problems, able to avoid duplication of efforts and to exploit existing resources to their best.

The paper by Gonzalez-Beltran, Neumann et al. "The Risa R/Bioconductor package: integrative data analysis from experimental metadata and back again" [14] presents a simple, effective, long awaited package that is a crucial tool for bridging data curation and data analysis, a bottleneck for research data management, with real world examples.

In scientific workflows, there often arise some patterns, so called "anti-patterns", that can lead to over-complicated design and may compromise, share and reuse of workflows. The paper "Distilling structure in Taverna scientific workflows: a refactoring approach" [15] by Cohen-Boulakia, Chen et al. presents a method to detect and remove "anti-patterns" in workflows automatically. The paper formally introduces two anti-patterns and illustrates the application of the method on more than 1,500 workflows from two distinct domains.

The paper "QTREDS: a Ruby on Rails-based platform for omics laboratories" [16] by Palla, Frau et al. describes a lightweight Laboratory Information Management System (LIMS) designed for the needs of a sequencing and genotyping laboratory. The system includes various functional blocks, including samples and reagents management, workflow generation and an articulated user interface.

In their paper "Guidelines for managing data and processes in bone and cartilage tissue engineering" [17], Viti, Scaglione et al. introduce a conceptual framework for bone/cartilage tissue engineering data. They present guidelines defining the minimum information necessary for describing an experimental study in this domain, as well as a devoted ontology, that is oriented both to cells and to chemical composition, morphology, and physical characterization of biomaterials involved in bone/cartilage tissue engineering research.

Text-mining applications for biomedical patents are relatively rare, although the size of patent collections is rapidly increasing. The paper "Development and tuning of an original search engine for patent libraries in medicinal chemistry" [18] by Pasche, Gobeill et al. presents an advanced search and retrieval engine for patents corpora. It also reports the results of extensive tests made to evaluate the impact of different search strategies on the performance of the search engine when applied to the most frequent search tasks performed in medical chemistry.

## List of abbreviations used

BITS: Bioinformatics Italian Society; GEO: Genome Expression Omnibus; ICT: Information and Communication Technologies; LIMS: Laboratory Information Management System; NETTAB: Network Tools and Applications in Biology; SeCo: Search Computing; SNP: Single Nucleotide Polymorphism; URI: Uniform Resource Identifier.

## Competing interests

The authors declare that they have no competing interests.

## Authors' contributions

All authors discussed and agreed about the organization of the paper. PR wrote the paragraphs related to the NETTAB Workshops, while MM contributed to the description of the rationale of NETTAB 2012 and the related topic description. Each author wrote some of the presentations of papers. All authors read and agreed on the final version of the paper.

## Declarations

Publication of this article has been funded by the IRCCS AOU San Martino IST.

This article has been published as part of *BMC Bioinformatics *Volume 15 Supplement 1, 2014: Integrated Bio-Search: Selected Works from the 12th International Workshop on Network Tools and Applications in Biology (NETTAB 2012). The full contents of the supplement are available online at http://www.biomedcentral.com/bmcbioinformatics/supplements/15/S1.
